# Comparison of *Candida antarctica* Lipase B Variants for Conversion of ε-Caprolactone in Aqueous Medium—Part 2

**DOI:** 10.3390/polym10050524

**Published:** 2018-05-14

**Authors:** Heidi Höck, Stefan Engel, Simone Weingarten, Helmut Keul, Ulrich Schwaneberg, Martin Möller, Marco Bocola

**Affiliations:** 1 DWI—Leibniz Institute for Interactive Materials and Institute of Biotechnology, RWTH Aachen University, Forckenbeckstraße 50, D-52056 Aachen, Germany; H.Hoeck@biotec.rwth-aachen.de (H.H.); Simone.weingarten@rwth-aachen.de (S.W.); U.Schwaneberg@biotec.rwth-aachen.de (U.S.); 2 DWI—Leibniz Institute for Interactive Materials and Institute of Technical and Macromolecular Chemistry, RWTH Aachen University, Forckenbeckstraße 50, D-52056 Aachen, Germany; Engel@dwi.rwth-aachen.de (S.E.); Keul@dwi.rwth-aachen.de (H.K.)

**Keywords:** *Candida antarctica* lipase B, enzymatic ring-opening polymerization, enzyme engineering, microgel, immobilization

## Abstract

Enzyme-catalyzed ring-opening polymerization of lactones is a method of increasing interest for the synthesis of polyesters. In the present work, we investigated which changes in the structure of *Candida antarctica* lipase B (CaLB) shift the catalytic equilibrium between esterification and hydrolysis towards polymerization. Therefore, we present two concepts: (i) removing the glycosylation of CaLB to increase the surface hydrophobicity; and (ii) introducing a hydrophobic lid adapted from *Pseudomonas cepacia* lipase (PsCL) to enhance the interaction of a growing polymer chain to the elongated lid helix. The deglycosylated CaLB (CaLB-degl) was successfully generated by site-saturation mutagenesis of asparagine 74. Furthermore, computational modeling showed that the introduction of a lid helix at position Ala148 was structurally feasible and the geometry of the active site remained intact. Via overlap extension PCR the lid was successfully inserted, and the variant was produced in large scale in Pichia pastoris with glycosylation (CaLB-lid) and without (CaLB-degl-lid). While the lid variants show a minor positive effect on the polymerization activity, CaLB-degl showed a clearly reduced hydrolytic and enhanced polymerization activity. Immobilization in a hydrophobic polyglycidol-based microgel intensified this effect such that a higher polymerization activity was achieved, compared to the “gold standard” Novozym^®^ 435.

## 1. Introduction

Biodegradable polymers have gained strong interest due to their advantages for medical applications and tissue engineering [1,2,3]. Biodegradable polyesters (PES) can be synthesized by enzyme-catalyzed ring-opening polymerizations (eROP), which benefits from mild reaction conditions and high control of enantio-, chemo-, and regioselectivity [4,5]. In contrast to the synthesis of PES via eROP, conventional chemical ROP performed with metal- or organocatalysts requires a purification step to reduce catalytic remains in the obtained polyester. Moreover, polymers for medical applications must be free of any metal traces and organic solvents; therefore, enzymatic polymerization is very advantageous in these applications. The first eROP was performed by two independent groups in 1993 using *Porcine pancreatic* lipase, *Pseudomonas fluorescens* lipase and *Candia cylindracea* lipase as biocatalyst [6,7].

**Figure 1:**
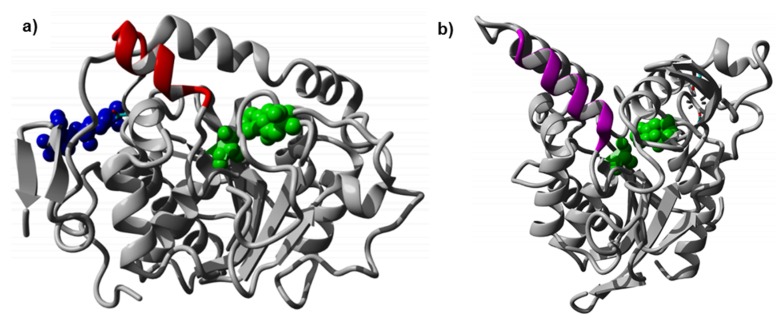
(**a**) Cartoon representation of CaLB (PDB ID: 1TCA) with active site residues Ser105, His224, Asp187 in green and glycosylation site Asn74 with two NAG residues in blue. The small lid helix flanking the binding site is colored red. (**b**) Cartoon representation of PsCL (PDB ID: 3LIP) with active site residues Ser87, His286, Asp264 in green. The large lid helix flanking the binding site is colored magenta.

Another widely used lipase in aminolysis, transesterification and enzymatic ROP of lactones in organic solvents is *Candida antarctica* lipase B (CaLB) [8,9]. This lipase is commercially known as Novozym^®^ 435, where it is immobilized on a macroporous acrylic resin. Structurally, CaLB belongs to the α/β hydrolase-fold family, represented by a conserved core structure with an eight-stranded, mostly parallel twisted β-sheet, flanked by two α-helices [10]. CaLB consists of 317 amino acids and has a molecular weight of 33 kDa. The catalytic triad is formed by Ser105, His224 and Asp187 (Figure 1a). Asparagine linked glycosylation increases the polarity of the protein surface and supports solubility in aqueous environment. CaLB has one *N*-glycosylation site at position Asn74 [10]. The CaLB surface is divided into patches that have a predominant hydrophobic nature near the lipid binding site and a hydrophilic nature at the back of the enzyme, allowing an orientation at water-lipid interfaces. The hydrophilic glycosylation moiety is in the hydrophilic patch of the enzyme [11]. The two first sugar moieties of N-linked glycan of CaLB covers a hydrophobic patch of the protein surface formed by Val64, Met298 and Pro299, which can be seen in the crystal structure (Protein Data Bank ID (PDB ID): 1TCA) [10]. Literature shows successful expression of the CaLB Asn74Ser with removed *N*-glycosylation site in *Pichia pastoris* and *E. coli*. This deglycosylated variant showed a lower specific activity independent from the expression host [12].

Unlike most lipases, the entrance to the CaLB active site has no large helical lid covering the active site in water and does not exhibit a strong interfacial activation [13]. Even though the crystal structure was already published in 1994, only recently it was discovered that CaLB possesses a lid-like structure which has an open and a closed state [14]. The interfacial activation of lipases involve typically the opening of a long lid-helix, covering the active site in the absence of a hydrophobic interface, which was shown e.g., for *Pseudomonas cepacia* lipase [15,16]. Among others, PsCL and *Bacillus thermocatenulatus* contain a very complex lid structure with two coupled lid helices [17,18]. Mutations in the lid structure of different lipases have been shown to influence the enantioselectivity, thermostability and chain length specificity [19,20,21,22]. Skjøt and colleagues found that altering composition and length of the CaLB lid increased hydrolytic activity on ester substrates with C_α_ branching on the carboxylic side and increased enantioselectivity of racemic ethyl 2-phenylpropanoate hydrolysis (E > 50) [19].

In their natural aqueous environment lipases are known to hydrolyze fatty acid esters at the lipid-water interface with formation of fatty acids and glycerol. It was shown that in organic solvents or at the interface of water and a water insoluble substrate the lipase also catalyzes esterification reactions [7]. However, hydrolysis and esterification are in an equilibrium which is controlled by the water concentration in the environment of the lipase. Immobilization to hydrophobic supporting materials, such as the acrylic resin beads for Novozym^®^ 435, is a common method for shifting the preferred catalyzed reaction towards esterification. Investigations of alternative carriers such as nano- or microgels for immobilization of catalysts in general and enzymes in particular attracted more and more interest during the last years [23,24]. Hereby, the influence of the gel network on the enzymatic activity and the protection against solvents are important research issues among others [25,26,27]. Recently, we reported about the synthesis and immobilization of CaLB in tailormade microgels and showed the influence of the microgel polarity on the ring-opening polymerization activity of CaLB [28,29,30]. Besides our work Montanier and colleagues published their work on engineering of CaLB for polyester synthesis [31].

In this work we attempt to increase the polymerization activity in aqueous medium by engineering of the CaLB structure. Special emphasis is placed on the deglycosylation to increase the surface of CaLB as well as the introduction of a hydrophobic lid helix close to the active center. The obtained variants are further immobilized in hydrophobic microgels and the hydrolytic and polymerization activity of the free and immobilized forms are evaluated and compared.

## 2. Materials and Methods

All chemicals were of analytical grade or higher quality and purchased from Sigma-Aldrich (Hamburg, Germany), Applichem (Darmstadt, Germany), Carl Roth (Karlsruhe, Germany), Formedium (Norfolk, UK), or Invitrogen (Darmstadt, Germany). All enzymes were purchased from New England Biolabs GmbH (Ipswich, MA, USA), Fermentas GmbH (St. Leon-Rot, Germany) or Sigma-Aldrich (Hamburg, Germany). All PCRs were performed in a Thermal cycler (Mastercycler proS; Eppendorf, Hamburg, Germany). The PCR volume was always 50 μL. The amount of DNA in cloning experiments was quantified using a NanoDrop photometer (ND-1000, NanoDrop Technologies, Wilmington, DE, USA). Oligonucleotides were purchased from Eurofins MWG operon (Ebersberg, Germany). Plasmid extraction and PCR purification kits were purchased from Macherey-Nagel (Düren, Germany). Omega FLUOstar (BMG LABTECH, Ortenberg, Germany) was used for absorbance detection. Microtiter plates (Greiner Bio-One GmbH, Kremsmünster, Austria) were incubated in a Multitron II Infors shaker (Infors AG, Bottmingen, Switzerland). CaLB gene was synthesized by GeneArt (Regensburg, Germany).

### 2.1. Cloning of Candida antarctica Lipase B into the pYES2 Expression Vector and Transformation into Saccharomyces cerevisiae

CaLB gene (GenBank: Z30645.1) (codon optimized for *S. cerevisiae*) together with α-secretion factor (MF-α) was purchased from Life Technology (Carlsbad, CA, USA). For plasmid construction a PCR-directed recombination in vivo method was used [32]. The CaLB gene and the target plasmid were separately amplified via PCR. The CaLB gene was amplified from the vector via PCR with forward primer CaLB_fw (5′-AGCTCGCCTGTGGTCTC-3′) and reverse primer CaLB_rev (5′-TGGCGGCCGTTACTAGTGGATCCGAGCTCATGGGGTAACGATACCAGAAC-3′). The pYES2 was amplified from the vector via PCR with forward primer pYES_fw (5′-GAGCTCGGATCCACTAGTAACG-3′) and reverse primer pYES_rev (5′-CTCCTGATGTAGTGTGGCAG-3′). Methylated template DNA was then digested in both reactions with restriction enzyme *DpnI* (20 U, 37 °C, 16 h). Yeast transformation was performed with lithium acetate-based method [33]. Positive clones were initially selected on SC-U agar plates (synthetic complete; yeast nitrogen base, yeast synthetic drop-out medium supplements without uracil, 5 g/L ammonium sulphate and 2% glucose) lacking uracil as selection marker. To confirm the correct construct one colony was grown in 10 mL overnight culture in SC-U in 50 mL shake flasks (16 h, 30 °C, 70% humidity, 200 rpm). Plasmid extraction was performed with Plasmid Extraction Kit from Machery-Nagel (Düren, Germany) after cells disruption with glass beads. Transformation into *Escherichia coli* DH5α cells (purchased from Agilent Technologies; Santa Clara, CA, USA) was performed, to reach higher plasmid concentration suitable for sequencing [34]. From *E. coli* isolated plasmid was sequenced by Eurofins GmbH (Ebersberg, Germany).

### 2.2. Generation of Deglycosylated Variant by Site-Saturation Mutagenesis

To obtain all 20 amino acids at position 74, with minimal stop codons, degenerated NNK codons were used (N = A/T/G/C, K = G/T). NNK encodes all 20 amino acids with 32 codons. A modified QuikChange PCR protocol using pYES_CaLB plasmid as template was used [35]. The PCR mixture was Phusion HF buffer (50 mL; New England Biolabs, Ipswich, MA, USA) containing dNTPs (0.2 mm), template DNA (17–20 ng), and Phusion DNA polymerase (1 U). For each targeted amino acid position, the reaction mixture was split into two tubes, and forward (5′-CACCACCATTCATGTTGNNKGATACTCAAG-3′) or reverse primer (5′-CTTGAGTATCMNNCAACATGAATGGTGGTG-3′) (10 mM) was added to each reaction. PCR was first performed with the following program: 98 °C/30 s; 98 °C/10 s, 60 °C/30 s, 72 °C/180 s (3 cycles). The forward and reverse reaction products were combined, and PCR was continued: 98 °C/30 s; 98 °C/10 s, 60 °C/30 s, 72 °C/180 s (11 cycles); 72 °C/180 s. Methylated template DNA was then digested with *DpnI* (20 U, 37 °C, 16 h). The PCR products followed a purification with the PCR clean-up gel extraction kit (Machery-Nagel, Düren, Germany) and subsequent transformation in *S. cerevisiae* [33].

### 2.3. Library Transformation and Expression in MTP

After transformation of the SSM library positive clones were selected on SC-U agar plates. The 88 CaLB clones were transferred in SC-U medium (100 µL) in a 96-well microtiter plate (MTP; flat-bottomed, polystyrene plates; Greiner Bio-One GmbH, Kremsmünster, Austria), along with four wild type (wt) clones and four empty vector clone (controls). The cultures were incubated (16 h, 30 °C, 70% humidity, 900 rpm) in a microtiter plate shaker (Infors, Eisenach, Germany). The plates were stored as “master plates” at −80 °C after addition of sterile glycerol (100 µL, 30% *w*/*v*) to each well. For variant expression, the frozen master plate was replicated into a 96-well MTP containing SC-U Agar (150 µL per well) and cultivated (48 h, 30 °C, 70% humidity). The cells were replicated into a new 96-well MTP containing SC-U medium with 2% glucose (150 µL per well) and cultivated (24 h, 30 °C, 70% humidity, 900 rpm). The cultures were again replicated into new 96-well, deep well, polystyrene MTPs (Greiner Bio-One) containing SC-U medium containing 0.5% glucose and 1.5% galactose (500 µL per well) and incubated (72 h, 30 °C, 70% humidity, 900 rpm). CaLB containing supernatant was collected after centrifugation of the expression culture (Eppendorf 5804R/5810R, 4000 *g*, 20 min, 4 °C) and used for activity measurements.

### 2.4. para-Nitrophenyl Butyrate (pNPB) Assay in MTP Format for CaLB Activity Measurement

The 88 resulting clones were tested for hydrolytic activity (library coverage 93.6%) [36,37]. The lipase activity was determined by addition of phosphate buffer (90 µL, 100 mM, pH 7.5) to culture supernatant lipase solution (10 µL) and freshly prepared substrate solution (phosphate buffer (100 µL) containing *p*NPB (0.5 mM) and acetonitrile (10 *v*/*v*%) in each well. The release of *para*-nitrophenolate, the hydrolysis product, was measured by the absorption at 405 nm at room temperature over 8 min on the microtiter plate reader. Activity was calculated as units per milligram CaLB (1 unit was defined as the amount of CaLB releasing 1 µmol *p*NPB in 1 min).

### 2.5. Molecular Modelling of CaLB Lid Variant

A computational modelling of a CaLB-lid variant was made by insertion of 11 amino acids (Tyr129-Ile139, sequence: YDPTGLSSTVI), forming a long helix flanking the acid pocket, based on the lipase from *Pseudomonas cepacia* (PsC) X-ray structure (PDB ID: 5LIP) into the X-ray crystal structure of CaLB with bound chiral inhibitor after the short lid helix at position Ala148 [15,38]. The newly inserted residues were placed according to a structural alignment of CaLB and PsCL using the software Mustang as implemented in Yasara Structure Vers. 13.1.25. [39,40]. The built CaLB lid structure was protonated at pH 7.4 and subsequently relaxed by initial steepest descent and simulated annealing minimization (timestep 2 fs, atom velocities scaled down by 0.9 every 10th step) employing NOVA force field until convergence (energy improved by less than 0.05 kJ/mol per atom during 200 steps) was reached [41,42]. The minimized structure of the CaLB-lid variant was subsequently further relaxed using 2 ns of molecular dynamics simulation solvated in a TIP3P cubic water box (size: 72.08 × 67.17 × 56.03 Å, density 0.997 g/mL), neutralized with 0.9% NaCl at 298 K, using a 7.86 Å electrostatic cut off and Particle Mesh Ewald for long range electrostatics. The minimized CaLB-lid structure showed an elongated regular helix structure from Leu140-Pro151, compared to a shorter helix Ala141-Leu147 found in the original CaLB-wt structure.

### 2.6. Insertion of Engineered Lid Helix via Overlap Extension PCR

The insertion of 11 amino acids was introduced by overlap extension PCR. The whole vector pYES_CaLB was amplified with the primer CaLB-lid_fw (5′-GGTTTGTCTTCCACTGTTATTGCT*CCATCTGTTTGGCAACAAACTACTG*-3′) CaLB-lid_rev (5′-CAGTGGAAGACAAACCAGTTGGATCATA*AGCCAAAGCAT CCAATGGAC*-3′) in a two-step PCR. PCR template was digested with restriction enzyme *DpnI* (20 U) by applying 1 µL of enzyme directly into PCR mix and incubated 16 h at 37 °C. The PCR product was purified with the PCR clean-up gel extraction kit and subsequently transformed into *E. coli* DH5α. The plasmid was extracted and sequenced.

### 2.7. Cloning of Candida antarctica Lipase B into the pGAPz Expression Vector and Transformation into Picha pastoris

Plasmid extraction of synthetic gene and pGAPzαA was done with the plasmid DNA purification kit. The CaLB gene was amplified from the vector via PCR with forward primer pGAP_fw (5′-GCTGAAGCTGAATTCTTGCCATCTGGTTCTG-3′) and reverse primer pGAP_rev (5′-CACACTGGGTACCCGTTACTAGTGGATCCG-3′). The PCR product and pGAPzαA were digested using restriction enzymes *EcoRI* (100 U) and *KpnI* (100 U). After 20 min heat inactivation at 80 °C and purification of the specific DNA fragments with the PCR clean-up gel extraction kit, the digested CaLB gene and vector pGAPzαA were ligated using T4 DNA ligase (5 U) resulting in pGAP_CaLB. The plasmid construct was subsequently transformed into *E. coli* DH5α cells (purchased from Agilent Technologies; Santa Clara, CA, USA). The pGAP_CaLB was extracted from *E. coli* DH5α. About 200 ng plasmid DNA linearized by *AvrII* was mixed with 80 µL of competent cells, and then it was transformed into *Pichia pastoris* (SMD1168) cells (purchased Invitrogen GmbH, Karlsruhe, Germany) by electroporation conducted on Eppendorf Eporator (Eppendorf, Hamburg, Germany) according to the manufacturers instruction *Pichia pastoris* transformants via homologous recombination at the GAP promoter region between the transforming DNA and regions of homology within the *Pichia* genome. Positive clones were initially selected on YPDS plates containing 100 μg/mL Zeocin™ plates.

### 2.8. Screening for Pichia Pastoris Strain with Highest CaLB Expression

To determine the *Pichia pastoris* strain with highest lipase production due to multi-copy recombinants 10 colonies were transferred into 96-well flat-bottom microtiter plates containing 150 μL YPD medium supplemented with Zeocin™ (0.07 µM or 0.1 mg/mL) (master plate). After overnight cultivation in a microtiter plate shaker (30 °C, 70% humidity, 900 rpm), 150 μL main culture (YPD medium without antibiotics) were inoculated with 10 μL pre-culture (v-bottom MTP, expression plate). The master plate was stored at −80 °C after addition of 100 μL glycerol (30% (*v*/*v*)). Expression plates were cultivated for (24 h, 20 °C, 70% humidity, 900 rpm). After expression, v-bottom MTPs were centrifuged for cell harvesting (10 min, 4 °C, 4000 *g*). The supernatant was transferred into a new flat-bottom microtiter plate and used for activity measurement.

### 2.9. Large Scale Production of CaLB in Shake Flasks and Enzyme Purification

*Pichia pastoris* cells pre-grown on YPD agar plate solid medium were inoculated in 10 mL YPD medium and incubated (16 h, 30 °C, 70% humidity, 220 rpm) as a pre-culture. The main-culture was inoculated at OD_600_ of 0.2 and incubated (72 h, 20 °C, 70% humidity, 220 rpm). The supernatant containing the secreted enzyme was separated from the cells by centrifugation (Sorval RC 6; Thermo Fisher Scientific, Waltham, MA, USA) (30 min, 4 °C, 4000 *g*). Tris-acetate buffer (pH 7.2; 250 mM) was added to recovered supernatant in relation 1:10 and filtered with a glass fiber filter (pore size 0.45 µm; GE Healthcare, Chicago, IL, USA). For enzyme purification anion-exchange chromatography was employed, using the ÄKTApilot system (GE Healthcare). The column was packed with 100 mL resin (Fractogel TSK DEAE-650s, Merck, Darmstadt, Germany), a flow rate of 10 mL/min was selected; equilibration with 200 mL Tris-acetate buffer (pH 7.2; 25 mM); sample load: 2 L supernatant. The flow through containing the enzyme was collected. In a further purification step hydrophobic interaction chromatography was employed. The column was packed with 100 mL resin (Fractogel TSK Butyl 650 Size S, Merck, Darmstadt, Germany), a flow rate of 10 mL/min was selected; equilibration with 200 mL tris-acetate buffer (pH 7.2; 25 mM) adjusted to 20 mS/cm with ammonium acetate; sample load: 2 L supernatant adjusted to 20 mS/cm with ammonium acetate; wash: 200 mL Tris-acetate buffer (pH 7.2; 25 mM, 20 mS/cm) and 100 mL ammonium acetate (0.3 M); elution with a gradient from 0.3 M ammonium acetate to water. The sample was lyophilized yielding 100 mg/2000 mL CaLB.

### 2.10. Thermal Resistance

The thermal resistance was determined by evaluation of the thermal activity after heat incubation. Therefore 15 µL of a 0.03 mg/mL lipase solution in phosphate buffer (0.1 M, pH 7.5) was heated in a 96-well PCR plate in a Mastercycler proS (Eppendorf, Hamburg, Germany). After preheating to a starting temperature of 40 °C the columns of the MTP were heated for 10 min at 39.9, 40.4, 41.7, 43.5, 45.8, 48.4, 51.1, 53.8, 56.2, 58.1, 59.5 and 60.1 °C and were immediately cooled down to 8 °C afterwards. The lipase activity was assayed in quadruplicate at room temperature as described above.

### 2.11. MALDI-TOF

MALDI-TOF (Matrix Assisted Laser Desorption Ionization, Time-of-Flight) mass spectrometry was performed on a Bruker ultrafleXtreme (Billerica, MA, USA) equipped with a 337 nm smartbeam laser in the reflective mode. Solutions of Super-DHB (sDHB) (10 µL of 50 mg/mL) and analyte (10 µL of 5 mg/mL) in TA50 (Acetonitrile/H_2_O/TFA 1:1:0.002) were mixed and 1 µL thereof was applied on the sample plate. Spectra were measured in the reflective mode and 5000 laser shots with 38% laser power were collected. Proteine Standard II was used for calibration.

## 3. Results and Discussion

Recently, our groups showed the activity effect of CaLB immobilization to tailored microgels of different polarity [30]. Providing a more hydrophobic environment in the microgel, the hydrolytic activity was reduced, and the polymerization activity was increased. Besides immobilization, engineering of the lipase is known to be a useful tool to influence its activity [19,31,43,44,45,46,47]. In the present work, two engineering approaches with CaLB are reported to increase the polymerization activity.

The first approach is the deletion of the *N*-glycosylation site. CaLB as a glycoprotein is bound to an oligosaccharide at position Asn74, which increases the surface hydrophilicity to improve water solubility. For our purpose, deglycosylation is intended to reduce the water content of the enzyme surface. Furthermore, it increases the solubility in a more hydrophobic medium, which is also important for the immobilization into hydrophobic microgels.

The second approach is the enlargement of the hydrophobic lid of CaLB, based on the PsCL lid structure. In theory, this reduces the water content close to the active site and further enhances hydrophobic interactions of the lid with the growing polymer chain, resulting in longer polymer chains. In the following sections the modelling and production of the deglycosylated CaLB and the CaLB-lid variants are described. Afterwards, the synergistic effect of both mutations was investigated by production of a deglycosylated CaLB-lid variant. Thermal resistance, molecular weight and hydrolytic activity of all produced lipases were analyzed. Furthermore, the immobilization of the CaLB variants to hydrophobic microgels and the polymerization ability was investigated, both of the free and the immobilized lipases entrapped in a tailor-made hydrophobic microgel.

### 3.1. Enzyme Variants with Increased Hydrophobicity

#### 3.1.1. Generation of Deglycosylated CaLB Variant

**Figure 2:**
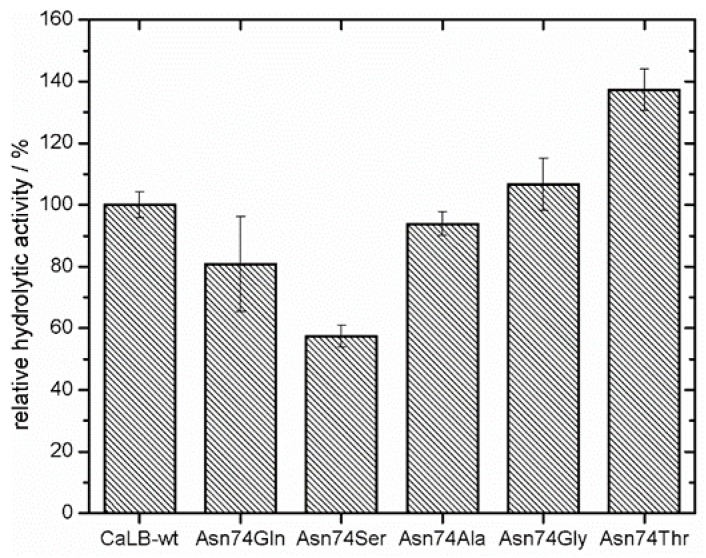
Relative hydrolytic activity of 5 best variants of CaLB Asn74 library measured from MTP supernatant.

The crystal structure of CaLB shows a glycosylation site at position Asn74 with linked *N*-glycan (see Figure 1a), which increases the polarity of the enzyme surface [10]. To screen all possible amino acid exchanges at position Asn74 a random mutagenesis library was generated. To obtain all 20 amino acids at each position, degenerated NNK codons were used (N = A/T/G/C, K = G/T), encoding all 20 amino acids with 32 codons. The library was successfully expressed in *S. cerevisiae* using the pYES expression system. The α-secretion factor in the pYES vector system allowed screening for hydrolytic activity in the medium without any purification step. The five clones with highest relative hydrolytic activity compared to CaLB-wt were sequenced, resulting in Asn74Gln, Asn74Ser, Asn74Ala, Asn74Gly and Asn74Thr mutations. In literature it is reported that the replacement of the glycosylation site with Ser has a negative effect on the catalytic activity of the lipase [12]. This stands in agreement with our results (Figure 2). For further experiments, Asn74Ala was chosen, since it showed approximately the same relative hydrolytic activity (94%; measured from MTP supernatant) as the wild type enzyme. Furthermore, alanine is a non-polar amino acid, which contributes to increase the hydrophobic surface.

#### 3.1.2. Simulation and Generation of CaLB-Lid Variants

**Figure 3:**
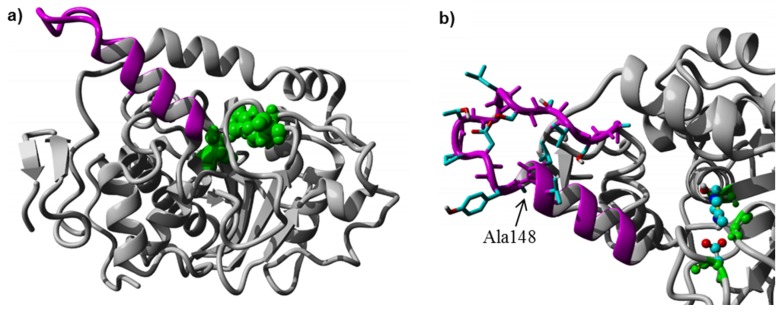
(**a**) Cartoon representation of CaLB-lid variant; Lid residues are colored in purple and active site residues in green; (**b**) Active site view showing the side chain orientation of the introduced residues from Ala148, elongating the acyl binding pocket. The side chains are shown in stick representation using atom colors (carbon: cyan, oxygen: red, nitrogen: blue, polar hydrogen: white, aliphatic hydrogens are omitted for clarity).

As mentioned above, CaLB has a small lid structure (Figure 1) in comparison to other lipases. In order to enlarge the binding pocket of CaLB, computational models of an insertion of the longer lid helix of PsCL, a lipase which showed the ability to form long chain polyesters in microemulsions [48], were constructed (Table 1). The structural model was used to analyze if the insertion of additional residues would be sterically and energetically feasible, without distorting the lid helix and the surrounding CaLB fold. The 3D-model was constructed and minimized according to the procedure described in methods section. The final relaxed model of CaLB with inserted PsCL lid helix residues after Ala148 (Figure 3a) revealed that the elongation of the lid helix was structurally feasible, and the H-bond network of the helix and the surrounding active site geometry remained intact, having a low RMSD of 0.82 Å compared to the X-ray structure of CaLB (PDB ID: 1TCA). The alternative model incorporating the complete 15 residue PsCL helix after Gly135 was structurally and energetically less favorable (data not shown) and the inserted PsCL-helix structure was not stable after simulated annealing minimization performed using the standard Yasara minimization experiment. The lid insertion after Ala148 was structurally more stable and energetically feasible. The insertion of 9 additional residues from PsCL and exchange of Ser150 to Ile introduced a 15 Å long hydrophobic patch, ranging from Leu147 to Trp155 at the tip of the elongated helix-turn motif (Figure 3b). The designed lid insertion after Ala148 was successfully introduced via overhang extension PCR. The lid construct was generated with both glycosylated (CaLB-lid) and deglycosylated (CaLB-degl-lid) variant.

#### 3.1.3. Production, Purification and Characterization of CaLB Variants

For large scale production and purification of CaLB-wt, CaLB-degl, CaLB-lid, CaLB-degl-lid the constructs were cloned into *Pichia pastoris* using pGAPz vector. The lipase was secreted in the medium and purified using IEX chromatography and HIC chromatography. The yield was approximately 50 mg freeze dried enzyme per 1000 mL YPD culture medium.

**Figure 4:**
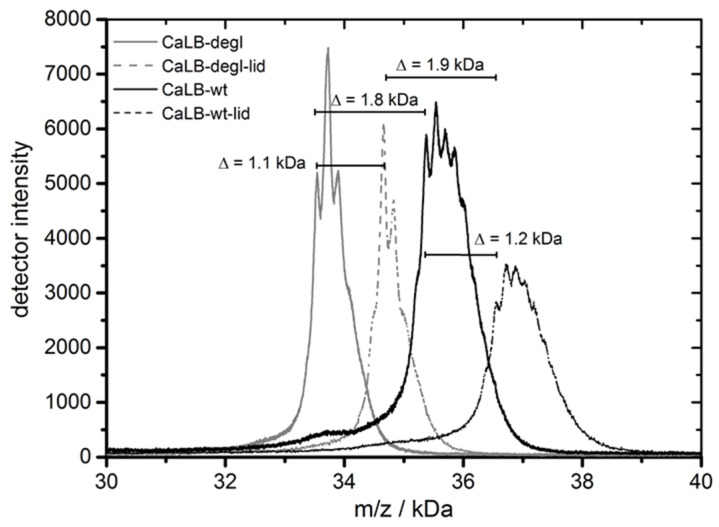
MALDI-TOF MS of CaLB variants (grey: CaLB-degl, grey (dashed): CaLB-degl-lid, black: CaLB-wt, black (dashed): CaLB-lid).

Characterization of the four above introduced CaLB variants was performed with the purified enzymes in terms of their molecular weight and thermal resistance. The MALDI-TOF spectrum (Figure 4, also Table S1) showed the molecular weight distributions ranging from 33.7 kDa for CaLB-degl to 36.8 kDa for CaLB-lid. The comparison of CaLB-wt vs. CaLB-degl and CaLB-lid vs. CaLB-degl-lid showed that the molecular weight of the oligosaccharide is about 1.8 kDa. This corresponds well to a (Man)_10_-GlcNAc moiety (molecular weight 1842 g/mol) found in secreted human lipase *N*-glycosylation in *P. pastoris* expression [49]. The change of the lid structure revealed a weight difference of 1.1 kDa, which was in compliance with the expected weight of the 11 inserted amino acids.

**Figure 5:**
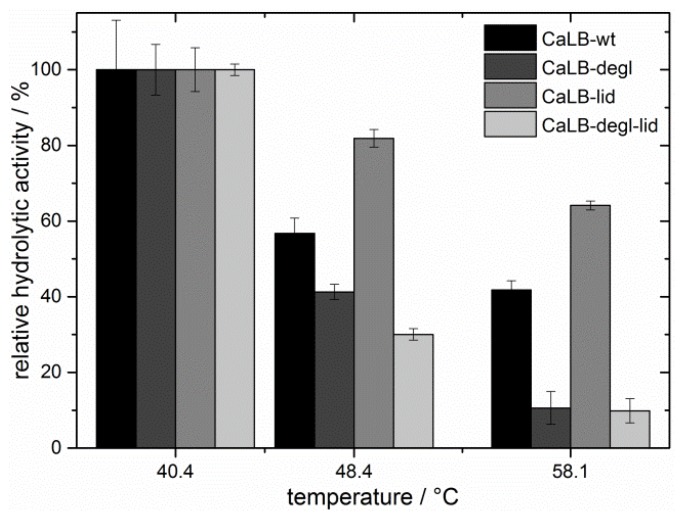
Relative hydrolytic activity after 10 min incubation at 40.4 °C, 48.4 °C and 58.1 °C for the CaLB-variants.

The thermal resistance was investigated by analyzing the residual hydrolytic activity of the lipases after incubation at different temperatures in a range of 40 to 60 °C (Figure 5). As expected, the residual hydrolytic activity of the deglycosylated variants decreased strongly with increasing temperature [50]. At 48 °C CaLB-degl and CaLB-degl-lid lost more than 50% of their initial activity and were almost inactive at 58 °C. CaLB-wt, in contrast, remained active, showing a relative activity of 42% at 58 °C. Surprisingly, an even higher thermal resistance was found for the CaLB-lid variant, showing more than 60% of relative hydrolytic activity at high temperatures (58 °C). Based on these results, the temperature for the following esterification experiments was chosen to be 45 °C, where the lipases are active to a reasonable degree.

### 3.2. Hydrolytic and Polymerization Activity

#### 3.2.1. Immobilization of CaLB Variants in Polyglycidol Based Microgels

**Scheme 1:**
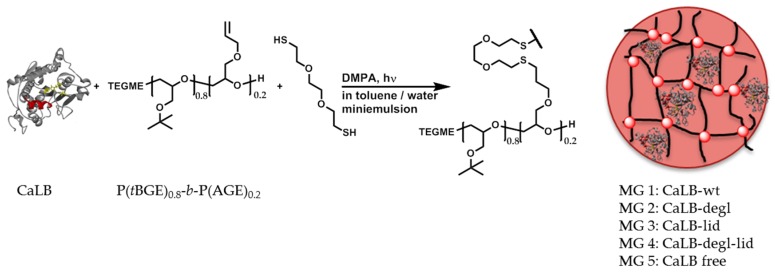
Synthetic route for the preparation of hydrophobic microgels using P(*t*BGE)_0.8_-*b*-P(AGE)_0.2_. CaLB-wt, CaLB-degl, CaLB-lid and CaLB-degl-lid were entrapped in situ during the microgel synthesis.

The immobilization of CaLB in polyglycidol-based microgels with controlled polarity was described by our group recently [28,29,30]. An efficient and elegant way to physically entrap the lipase in the microgel network was made by crosslinking of functionalized polyglycidol prepolymers in miniemulsion in the presence of CaLB-wt. An increased polymerization activity of CaLB-wt in dependence of the hydrophilic-lipophilic balance of the surrounding microgel was achieved [30]. This entrapping method (Scheme 1) was also adopted in this work and the four purified variants, CaLB-wt, CaLB-degl, CaLB-lid and CaLB-degl-lid, were immobilized in a hydrophobic microgel composed of P(*t*BGE)_0.8_-*b*-P(AGE)_0.2_ (Supplementary Information, Sections S3 and S4).

Table 2 shows the hydrodynamic radii and dispersities for the corresponding microgels. For all variants homogenous microgel dispersions were obtained with a hydrodynamic radius around 100 nm and a dispersity of 0.1. However, for both lid variants, a precipitate remained in the dialysis tubes after purification, which denotes partial denaturation of the lipase.

#### 3.2.2. Hydrolytic versus Polymerization Activity

**Scheme 2:**
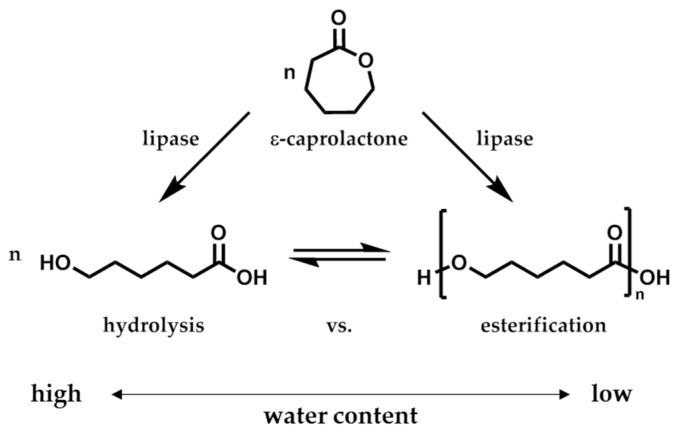
Catalytic role of the lipase in ring-opening polymerization of ε-CL: Equilibrium of hydrolysis and esterification depending on the water content.

The enzymatic activity of CaLB is described by the hydrolytic and esterification activity, here more precisely as polymerization activity (Scheme 2). While the hydrolytic activity is determined by the *p*NPB assay, the polymerization activity was determined by the polymerization of ε-caprolactone (ε-CL) in aqueous toluene solution. Since both activities are connected to each other due to the hydrolytic-esterification equilibrium, a conclusion of the enzyme performance had always to be drawn after evaluation of both activity values.

**Figure 6:**
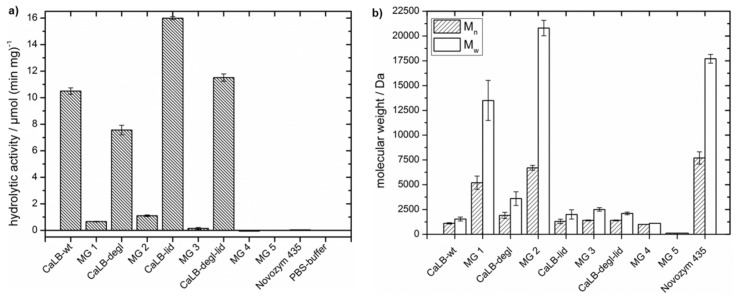
(**a**) Hydrolytic activity determined by *p*NPB-assay and (**b**) polymerization activity of CaLB-wt, CaLB-degl, CaLB-lid and CaLB-degl-lid both free and immobilized in hydrophobic microgels (MG 1 with CaLB-wt, MG 2 with CaLB-degl, MG 3 with CaLB-lid, MG 4 with CaLB-degl-lid). As a reference the enzyme free microgel MG 5, Novozym^®^ 435 and PBS buffer are shown. All experiments were performed in triplicate.

First, in Figure 6a the hydrolytic activity per milligram of purified enzyme at room temperature was plotted for the free and immobilized CaLB variants. Comparison of the free lipases showed that the hydrolytic activity decreased with the deglycosylation of the wild-type. This is consistent with the theoretical concept, that the increased surface hydrophobicity after deglycosylation reduces the water content close to the lipase. Further both CaLB-wt and CaLB-degl showed a strong reduction of hydrolytic activity when immobilized in hydrophobic microgels (MG 1 and MG 2), however, their hydrolytic activities did not differ significantly. The low hydrolytic activity of Novozym^®^ 435 is also shown in Figure 6a and emphasized the strong influence of the acrylic resin beads in the commercial, immobilized form of CaLB. The control MG 5, a lipase free microgel, showed no hydrolytic activity.

Even though again the trend for a reduced hydrolytic activity by deglycosylation is seen for the lid variants, the overall hydrolytic activity is higher than for CaLB-wt and CaLB-degl. However, immobilization of the lid variants gave lower hydrolytic activities than for CaLB-wt and CaLB-degl, where the CaLB-degl-lid variant showed no hydrolytic activity (MG 3 and MG 4). This could be explained by complete suppression of hydrolytic catalysis or by a denaturation of the lipase, needing further evaluation (see also Table 3).

The polymerization activity was determined by polyesterification of ε-CL in aqueous toluene (water content approx. 300 ppm; see Supplementary Information, Section S6). The obtained oligo- or polyesters were analyzed by SEC and NMR spectroscopy, respectively, and compared to Novozym^®^ 435 and microgel MG 5 (Figure 6b and Table 3). The results of MG 1 with CaLB-wt, published recently by our group, are shown again here for comparison [30]. Here it was observed, that the molecular weight increased from 1100 to 5200 Da after immobilization in the hydrophobic microgel. With the engineered, more hydrophobic, CaLB-degl variant polycaprolactone with a molecular weight of 1900 Da for the free lipase and 6700 Da for MG 2 was obtained. The broad molecular weight distribution (*M*_w_ = 3600 and 20,800 Da) shows also high molecular weight fractions in the polymer (compare Supplementary Information, Figure S6). Furthermore, the conversion of ε-CL to oligomers *C*_oligo_ was increased for the free deglycosylated variant by 16% to 29% of oligomeric product and for immobilized CaLB-degl by 5% to 91% after 40 h of reaction time (calculation of *C*_oligo_ is explained in Section S5 of the Supplementary Information). These results showed that deglycosylation is a successful tool to push the equilibrium towards esterification by reducing the water content at the lipase surface. This effect was enhanced by immobilization to microgels, which further provide a hydrophobic environment with reduced water content. It needs to be pointed out, that a higher polymerization activity is obtained than for the “gold standard” Novozym^®^ 435.

On the other hand, the lid variants demonstrate an unexpected trend. While the CaLB-lid shows a slightly higher conversion *C*_oligo_ (36%) and a molecular weight of P(ε-CL) of 1300 Da (*Ð* 1.5), the deglycosylation of CaLB-lid did not show an effect similar to the one observed for CaLB-degl; presenting the same polymerization activity as the one observed for CaLB-wt. Furthermore, the immobilization of the lid variants to microgels decreased the polymerization activity; being more pronounced for CaLB-degl-lid, where the conversion was reduced to 3% and the polymer molecular weight reached only 1000 Da after 40 h. These results corroborate the (partially) inactivation of the lid variants during the microgel preparation and polymerization experiments in a hydrophobic solvent. A possible explanation is an enhanced interaction of the hydrophobic lid with the hydrophobic solvent and microgel, leading to inhibition and therefore inaccessibility of the active center for the monomer.

## 4. Conclusions

In the present study we described the modelling, cloning and production of rationally engineered hydrophobic CaLB variants as well as their immobilization to hydrophobic microgels and the resulting changes in hydrolytic and polymerization activity. First, by exchange of asparagine at position 74 by alanine we were able to remove the *N*-glycosylation of CaLB reducing the surface hydrophilicity. Furthermore, a model of a CaLB-lid variant was constructed, where an elongated hydrophobic helix-turn motif of 11 amino acids, which was adapted from PsCL, was introduced close to the active site. Active CaLB variants without glycosylation site and/or exchanged lid helix were successfully produced and characterized by MALDI-TOF experiments. The CaLB variants were immobilized to hydrophobic, polyglycidol-based microgels using the recently published entrapping method [30]. Investigations of the enzymatic activity showed a reduced hydrolytic activity for the free deglycosylated variants compared to their glycosylated analogues. Simultaneously, the polymerization activity was increased by a factor of 1.7. This effect was even more pronounced for immobilized CaLB-degl; here a conversion of 91% to polycaprolactone with a molecular weight *M_n_* 6900 Da and a broad molecular weight distribution was obtained. This exceeds the polymerization ability of the “gold standard” Novozym^®^ 435 under the same reaction conditions. The lid variants having an enlarged hydrophobic lid at the active site also showed slightly enhanced polymerization activity and in their immobilized forms a huge decrease in hydrolytic activity. Polymerization experiments in hydrophobic microgels in contrast showed no enhanced activity, denoting inhibition of the lipase during the immobilization process.

## Figures and Tables

**Table 1:** Insertion of a lid helix on amino acid level; **bold**: original short helix, *italic*: insertion.

**#Pos**	**142**	**143**	**144**	**145**	**146**	**147**	**148**	**I**	**II**	**III**	**IV**	
CaLB-wt	**Gly**	**Pro**	**Leu**	**Asp**	**Ala**	**Leu**	**Ala**	-	-	-	-	
CaLB-lid	**Gly**	**Pro**	**Leu**	**Asp**	**Ala**	**Leu**	**Ala**	*Tyr*	*Asp*	*Pro*	*Thr*	
**#Pos**	**V**	**VI**	**VII**	**VIII**	**IX**	**149**	**150**	**151**	**152**	**153**	**154**	**155**
CaLB-wt	-	-	-	-	-	**Val**	**Ser**	Ala	Pro	Ser	Val	Trp
CaLB-lid	*Gly*	*Leu*	*Ser*	*Ser*	*Thr*	*Val*	*Ile*	Ala	Pro	Ser	Val	Trp

**Table 2:** Hydrodynamic radius *R*_h_ and dispersity *Ð* measured by DLS for microgels loaded with CaLB-wt, CaLB-degl, CaLB-lid and CaLB-degl-lid respectively and a CaLB free microgel as reference.

	Lipase	*R*_h,DLS_/nm	*Ð*
MG 1	CaLB-wt	101	0.10
MG 2	CaLB-degl	98	0.10
MG 3	CaLB-lid	102	0.09
MG 4	CaLB-degl-lid	107	0.09
MG 5	-	91	0.13

**Table 3:** Hydrolytic and polymerization activity of free and immobilized CaLB variants. Evaluation of hydrolytic activity by *p*NPB assay in TEA buffer and of polymerization activity by ε-CL polymerization in toluene (H_2_O content approx. 300 ppm).

No.	Catalyst	Hydrolytic Activity/µmol(min∙mg)^−1^	Polymerization Activity			
			*C*_oligo_/% ^1^	*M*_n_/Da ^2^	*M*_w_/Da ^2^	*Ð* ^2^
1 [30]	CaLB-wt	10.49	13	1100	1500	1.4
2 [30]	MG 1	0.66	86	5200	13,500	2.6
3	CaLB-degl	7.57	29	1900	3600	1.9
4	MG 2	1.10	91	6700	20,800	3.1
5	CaLB-lid	15.99	36	1300	2000	1.5
6	MG 3	0.15	45	1400	2500	1.8
7	CaLB-degl-lid	11.51	10	1400	2100	1.5
8	MG 4	0	3	1000	1100	1.1
9 [30]	Novozym^®^ 435	0.04	96	7700	17,700	2.3
10 [30]	MG 5	0	0	monomer	-	-

^1^ by NMR in CDCl_3_; ^2^ by SEC in THF.

## Supplementary Materials

The following are available online at http://www.mdpi.com/2073-4360/10/5/524/s1, Figure S1. ^1^H NMR spectrum (in CDCl_3_) of P(*t*BGE)_0.8_-*b*-P(AGE)_0.2_, Figure S2. ^13^C NMR spectrum (in CDCl_3_) of P(*t*BGE)_0.8_-*b*-P(AGE)_0.2_, Figure S3. SEC trace (in THF) of P(*t*BGE)_0.8_-*b*-P(AGE)_0.2_, Figure S4. ^1^H NMR spectrum in CDCl_3_ of the polymerization of ε-CL showing the signals for the ε-CL monomer (a), the hydrolysis product 6-hydroxyhexanoic acid (b) and the poly/oligo(ε-CL) (c), Figure S5. (a) SEC trace in THF and (b) ^1^H NMR spectrum in CDCl_3_ for the enzymatic polymerization of ε-CL with CaLB-degl, Figure S6. (a) SEC trace in THF and b) ^1^H-NMR spectrum in CDCl_3_ for the enzymatic polymerization of ε-CL with MG 2, Figure S7. (a) SEC trace in THF and (b) ^1^H-NMR spectrum in CDCl_3_ for the enzymatic polymerization of ε-CL with CaLB-wt-Lid, Figure S8. (a) SEC trace in THF and (b) ^1^H NMR spectrum in CDCl_3_ for the enzymatic polymerization of ε-CL with MG 3, Figure S9. (a) SEC trace in THF and (b) ^1^H NMR spectrum in CDCl_3_ for the enzymatic polymerization of ε-CL with CaLB-degl-lid, Figure S10. (a) SEC trace in THF and (b) ^1^H NMR spectrum in CDCl_3_ for the enzymatic polymerization of ε-CL with MG 4. Table S1. Molecular weights of CaLB-variants determined by MALDI-TOF spectroscopy, Table S2. Weighed portions of starting materials for the synthesis of P(*t*BGE)-*b*-P(AGE)-based microgels MG 1 to 5, Table S3. Enzymatic polymerization with 1 mg CaLB-degl, Table S4. Enzymatic polymerization with MG 2, Table S5. Enzymatic polymerization with 1 mg CaLB-wt-lid, Table S6. Enzymatic polymerization with MG 3, Table S7. Enzymatic polymerization with 1 mg CaLB-degl-Lid, Table S8. Enzymatic polymerization with MG 4.
