# Supplementary material for: Comparison of Candida antarctica Lipase B Variants for Conversion of ε-Caprolactone in Aqueous Medium—Part 2
Source: Polymers (Basel). 2018 May 14;10(5):524. doi: 10.3390/polym10050524 (PMC6415414; doi:10.3390/polym10050524)
Supplement: Supplementary file 1 [file polymers-10-00524-s001.pdf]

Supplementary Information

Comparison of *Candida antarctica* Lipase B Variants for Conversion of ε-Caprolactone in Aqueous Medium - Part 2

Heidi Höck [1,†], Stefan Engel [2,†], Simone Weingarten [1], Helmut Keul [2], Marco Bocola [1,*], Martin Möller [2,*] and Ulrich Schwaneberg [1]

[1] DWI – Leibniz Institute for Interactive Materials and Institute of Biotechnology, RWTH Aachen University, Forckenbeckstraße 50, D-52056 Aachen, Germany
[2] DWI – Leibniz Institute for Interactive Materials and Institute of Technical and Macromolecular Chemistry, RWTH Aachen University, Forckenbeckstraße 50, D-52056 Aachen, Germany
* Correspondence: M.Bocola@biotec.rwth-aachen.de; Moeller@dwi.rwth-aachen.de, Tel.: +49-241-80-23302
† Authors contributed equally to this work

1 Material and Methods 2
2 MALDI-TOF of CaLB variants 3
3 Synthesis of P(*t*BGE)$_{0.8}$-*b*-P(AGE)$_{0.2}$ 3
4 Synthesis of Polyglycidol Based Microgels with in situ Entrapment of CaLB Variants 5
5 Determination of C_{oligo} 5
6 Enzymatic ROP of ε-CL with Free and Immobilized CaLB Variants 6

1 Material and Methods

ε-Caprolactone (99%, Alfa Aesar, Karlsruhe, Germany), allyl glycidyl ether (AGE, >99%, Sigma Aldrich Chemie GmbH, Steinheim, Germany), *tert*-butyl glycidyl ether (*t*BGE, 99%, Sigma Aldrich Chemie GmbH, Steinheim, Germany), toluene (VWR Chemicals, Fontenay-sous-Bais, France), caesium hydroxide (96%, Alfa Aesar, Karlsruhe, Germany), 2,2-dimethoxy-2-phenylacetophenone (DMPA, 99%, Sigma Aldrich Chemie GmbH, Steinheim, Germany), sodium dodecyl sulfate (SDS, Bio-Rad Laboratories GmbH, München, Germany), hexadecane (99%, Sigma Aldrich Chemie GmbH, Steinheim, Germany), 2,2′-(ethylenedioxy)diethanethiol (95%, Sigma Aldrich, Quimica, Toluca, Mexico), triethylene glycol monomethyl ether (TEGME, >97%, Merck, Darmstadt, Germany), calcium hydride (93%, Thermofisher Acros Organics, Geel, Belgium) were used as received. Toluene, *tert*-butyl glycidyl ether, allyl glycidyl ether and ε-caprolactone were dried over CaH_2 for 24 h and distilled under nitrogen atmosphere before use.

1.1 NMR spectroscopy

^{1}H- and ^{13}C-NMR spectra were recorded on a Bruker Avance III 400 spectrometer (400 MHz and 100 MHz, respectively; Bruker Corporation, Billerica, MA, USA) and are reported as follows: chemical shift δ (ppm) (multiplicity, number of protons, assignment). Chloroform ($CDCl_3$, δH = 7.26 ppm, δC = 77.0 ppm) was used as an internal standard. Chemical shifts are reported in ppm to the nearest 0.01 ppm for ^{1}H- and the nearest 0.1 ppm for ^{13}C-NMR.

1.2 SEC

Molecular weights (M_n and M_w) and molecular weight distributions ($Đ$) were determined by size-exclusion chromatography (SEC). The results were evaluated using the PSS WinGPC UniChrom software (Version 8.1.1). SEC analyses were carried out with tetrahydrofuran (THF, HPLC grade, Carl Roth, Karlsruhe, Germany) as eluent using a HPLC pump (PU-2080plus, Jasco, Gross-Umstadt, Germany) equipped with a refractive index detector (RI-2031plus, Jasco, Gross-Umstadt, Germany). The sample solvent contained 250 mg·mL^{-1} 3,5-di-*tert*-4-butylhydroxytoluene (BHT, ≥99%,Sigma Aldrich Chemie GmbH, Steinheim,, Germany) as internal standard. One pre-column (8 mm × 50 mm) and four SDplus gel columns (8 mm × 300 mm, SDplus, MZ Analysentechnik, Mainz, Germany) were applied at a flow rate of 1.0 mL·min^{-1} at 20 °C. The diameter of the gel particles measured 5 µm, the nominal pore widths were 50, 10^2, 10^3 and 10^4 Å. Calibration was achieved using narrow distributed poly(methyl methacrylate) standards (PSS Polymer Standards Service GmbH, Mainz, Germany) from 600 to 576 000 Da.

1.3 Dynamic light scattering

Dynamic light scattering (DLS) measurements were performed with a commercial laser light scattering spectrometer (ALV/DLS/SLS-5000) equipped with an ALV-5000/EPP multiple digital time correlator and laser goniometry system ALV/CGS-8F S/N 025 with a helium neon laser (Uniphase 1145P, output power of 22 mW and wavelength of 632.8 nm) as a light source. As solvent Milli Q water was used. The hydrodynamic radius of the particles was determined by cumulant analysis.

2 MALDI TOF of CaLB variants

Table S1. Molecular weights of CaLB-variants determined by MALDI-TOF spectroscopy.

lipase	m/z / kDa
CaLB-degl	33.7
CaLB-degl-lid	34.7
CaLB-wt	35.6
CaLB-wt-lid	36.8

3 Synthesis of P(*t*BGE)$_{0.8}$-*b*-P(AGE)$_{0.2}$

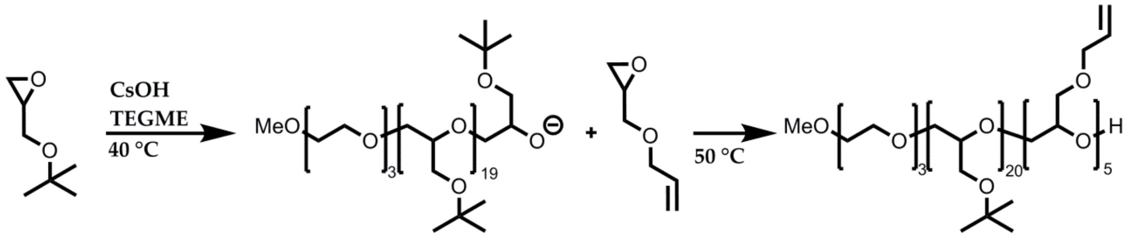

Caesium hydroxide (0.433 g, 2.58 mmol, 1/25 eq.) and triethylene glycol monomethylether (0.421 g, 2.58 mmol, 1/25 eq.) were dissolved in benzene (5 mL) and stirred at 60 °C for 30 min. The benzene/water azeotrope was removed by distillation at 90 °C and the residue was dried for another 3 h at 90 °C. *tert*-Butyl glycidyl ether (7.00 g, 53.77 mmol, 0.8 eq.) was added at room temperature and the reaction mixture was heated to 40 °C. After 17 h allyl glycidyl ether (1.49 g, 13.44 mmol, 0.2 eq.) was added and the temperature was increased to 50 °C. After 24 h and full conversion the polymerization was terminated by the addition of ethanol (1 mL). The polymer solution in ethanol was precipitated in cold water, filtered and dried in vacuum. The product was obtained as orange oil. **Yield:** 8.13 g (96%).

^{1}H-NMR (400 MHz, CDCl$_3$): δ = 5.85 (m, 1H, -OCH$_2$C*H*CH$_2$), 5.25-5.11 (dd, 2H, -OCH$_2$CHC*H*$_2$), 3.97 (d, 2H, -OC*H*$_2$CHCH$_2$), 3.63-3.35 (m, 10 H, -C*H*$_2$CHO-$_{(backbone)}$, -OCH$_2$C*H*O-$_{(backbone)}$, -O-CH$_2$CH$_{(backbone)}$C*H*$_2$O-), 3.35 (s, 3H, -O-C*H*$_{3(Initiator)}$), 1.14 (s, 9H, -O-C(C*H*$_3$)$_3$) ppm. ^{13}C-NMR (100 MHz, CDCl$_3$): δ = 135.0, 116.8, 79.2, 72.8, 72.3, 70.9, 70.1, 62.2, 59.1, 27.7 ppm.

SEC (THF): M_n = 3700 Da, $Đ$ = 1.05 (Figure S1-S3).

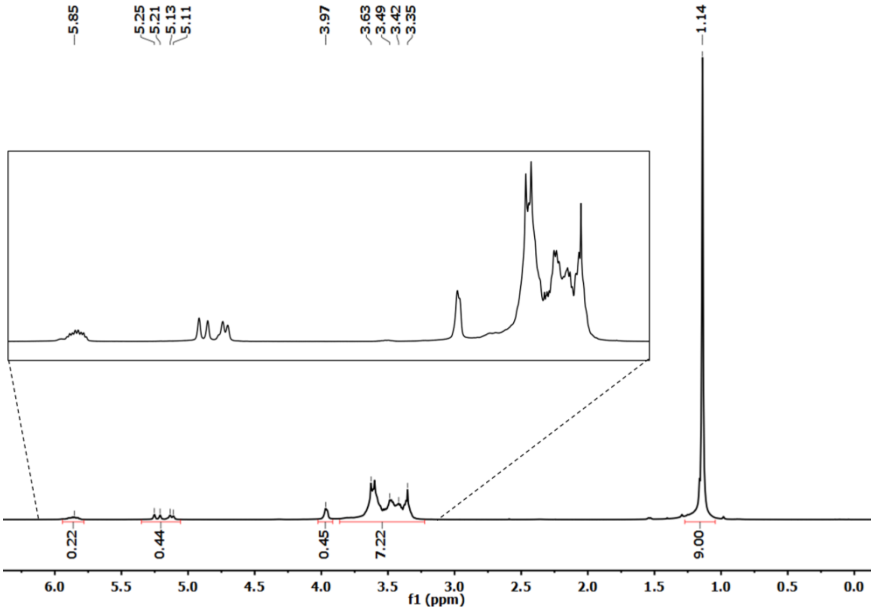

Figure S1. ^{1}H-NMR spectrum (in $CDCl_3$) of P(*t*BGE)$_{0.8}$-*b*-P(AGE)$_{0.2}$.

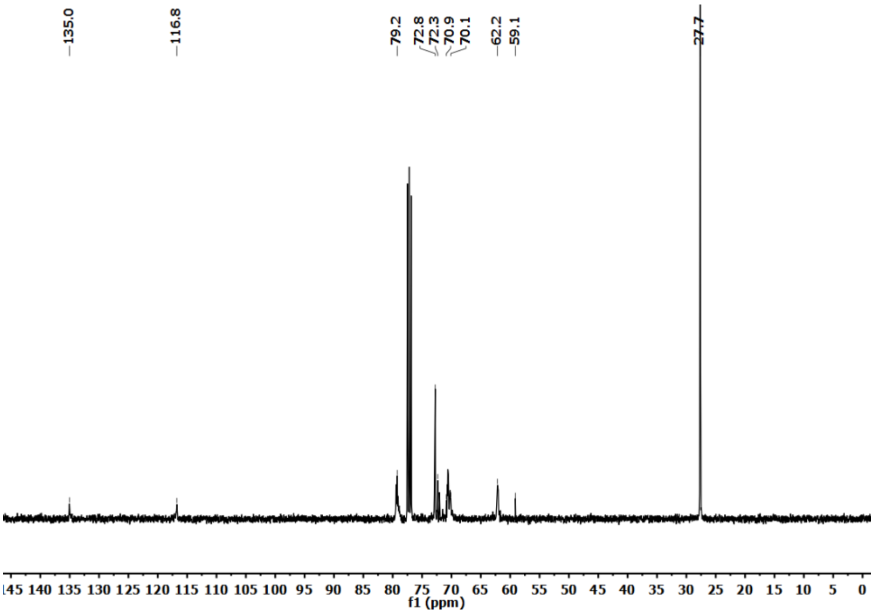

Figure S2. ^{13}C-NMR spectrum (in $CDCl_3$) of P(*t*BGE)$_{0.8}$-*b*-P(AGE)$_{0.2}$.

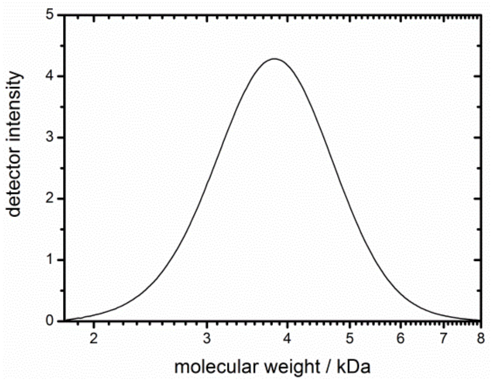

Figure S3. SEC trace (in THF) of P(*t*BGE)$_{0.8}$-*b*-P(AGE)$_{0.2}$.

4 Synthesis of Polyglycidol Based Microgels with in situ Entrapment of CaLB Variants

The procedure for the synthesis of CaLB loaded polyglycidol based microgels was reported in detail by our group [1]. Exemplarily the procedure for the preparation of MG 1 is explained. P(*t*BGE)$_{0.8}$-*b*-P(AGE)$_{0.2}$ (0.5 g, 0.79 mmol allyl groups, 1 eq.), free CaLB (5 mg), 2,2′-(ethylenedioxy)diethanethiol (0.079 g, 0.43 mmol, 0.55 eq.), hexadecane (0.04 g) and 2,2-dimethoxy-2-phenylacetophenone (0.018 g) were dissolved in toluene (1.78 g). A solution of sodium dodecylsulfate (0.0025 g) in water (8.0 g) was filtered and added to the organic phase. After a miniemulsion was prepared with a Branson Ultrasonifier 450 for 15 min (output control 3, duty cycle 50%) the emulsion was irradiated with a UV-LED cube (λ = 365 nm) for 2 h under stirring with a mechanical stirrer (1000 rpm). The microgel was purified by dialysis against water (molecular weight cutoff: 100 kDa). MG 2 - MG 5 were prepared analogue to MG 1 (Table S3).

Table S2. Weighed portions of starting materials for the synthesis of P(*t*BGE)-*b*-P(AGE) based microgels MG 1 to 5.

	**MG 1**	**MG 2**	**MG 3**	**MG 4**	**MG 5**
	CaLB-wt	CaLB-degl	CaLB-wt-lid	CaLB-degl-lid	CaLB-free
m(P(*t*BGE)-*b*-P(AGE)) / g	0.5	0.5	0.5	0.5	0.25
m(H_2O) / g	8.0	8.0	8.0	8.0	4.0
m(toluene) / g	1.78	1.78	1.78	1.76	0.88
m(dodecylsulfate) / g	0.0025	0.0025	0.0025	0.0025	0.002
m(hexadecane) / g	0.04	0.04	0.04	0.04	0.02
m(2,2′-(ethylenedioxy)diethanethiol) / g	0.079	0.075	0.075	0.075	0.038
m(2,2-dimethoxy-2-phenylacetophenone) / g	0.018	0.018	0.018	0.018	0.009
m(CaLB) / g	0.005	0.005	0.005	0.005	-

5. Determination of C_{oligo}

The conversion of ε-CL to oligomers (C_{oligo}) or polymers (C_{polym}) respectively is determined from the ^{1}H-NMR spectra (Figure S4) by using the discrete signals of the protons in γ–position for ε -CL, and the respective protons of 6-hydroxyhexanoic acid and the oligomer/polymer. While the signal at δ = 4.15 ppm (1a) is assigned to the ε-CL, the signal for the polymer/oligomer is found at δ = 3.97 ppm (1c) if the spectrum is measured in $CDCl_3$. The signal of the end group (1b) of both the oligomer and the 6-hydroxyhexanoic acid is found at a shift of δ = 3.54 ppm. Therefore the conversion C_{oligo} is calculated by

$$C_{oligo} = \frac{\int 1c}{\int 1a + \int 1b + \int 1c} \quad . \qquad (1)$$

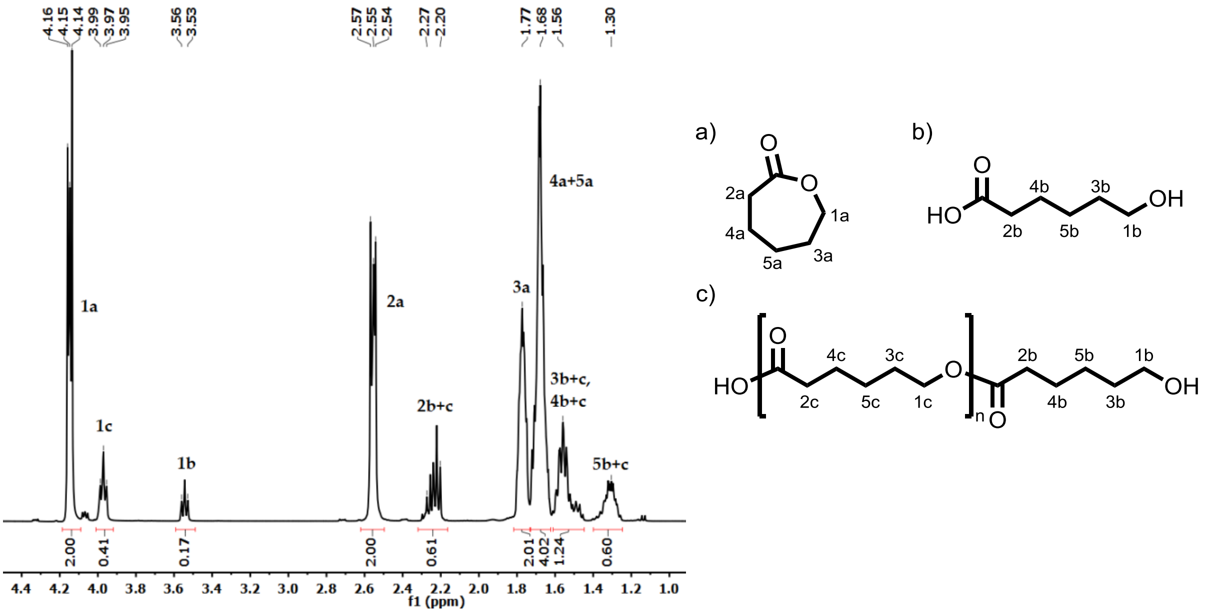

Figure S4. ^{1}H-NMR spectrum in $CDCl_3$ of the polymerization of ε-CL showing the signals for the ε-CL monomer (a), the hydrolysis product 6-hydroxyhexanoic acid (b) and the poly/oligo(ε-CL) (c).

6 Enzymatic ROP of ε-CL with Free and Immobilized CaLB Variants

In a 25 mL Schlenk tube CaLB (1 mg) was mixed under nitrogen atmosphere with anhydrous toluene (0.5 g), water saturated toluene (1.24 g) and ε-CL monomer (0.8 g). The solution was stirred for 46 h at 45 °C. The solvent was evaporated and the product was dried in vacuum. The product was analyzed by NMR and SEC without further purification.

In case of immobilized lipase, the microgels were dialyzed against THF and toluene (MWCO 10 kDa), before they were mixed with the solvent and monomer analogue to the free enzyme. The volume of the microgels was chosen in such, that 1 mg lipase was used for catalysis. All experiments were performed in triplicate and averaged. The respective weights of starting materials and product analytics are shown in the following tables. The NMR spectra and SEC traces are shown exemplarily in the corresponding figures below.

6.1 Weights and Results for free CaLB-degl

Table S3. Enzymatic polymerization with 1 mg CaLB-degl: Weights of anhydrous toluene, water saturated toluene and anhydrous ε-CL; the corresponding conversion to oligomers C_{oligo}, the molecular weight M_n and the polydispersity *Đ* were determined by ^{1}H-NMR (in $CDCl_3$) and SEC (in THF) respectively.

No.	anhydr. toluene / g	H_2O sat. toluene / g	ε-CL / g	total H_2O content / ppm	C_{oligo} / %	M_n / Da	*Đ*
1.1	0.72	2.53	0.83	285	42	2400	1.9
1.2	0.53	2.96	0.83	334	20	1600	1.7
1.3	0.52	2.72	0.82	338	26	1800	1.9
**Average**	**-**	**-**	**-**	**319**	**29**	**1900**	**1.9**

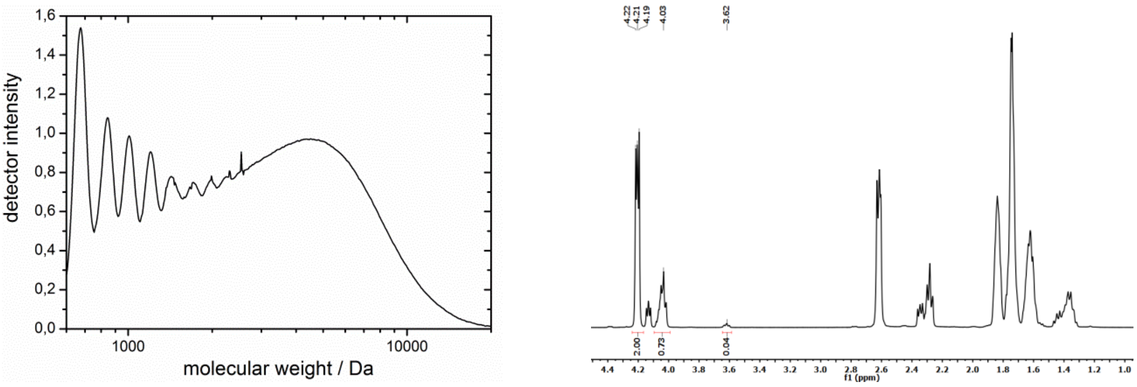

a) b)

Figure S5. a) SEC trace in THF and **b)** ^{1}H-NMR spectrum in $CDCl_3$ for the enzymatic polymerization of ε-CL with CaLB-degl (No. 1.3).

6.2 Weights and Results for MG 2 (CaLB-degl)

Table S4. Enzymatic polymerization with MG 2 ([CaLB-degl] = 0.45 wt/v): Weights of water saturated toluene, MG 2 and anhydrous ε-CL; the corresponding conversion to oligomers C_{oligo}, the molecular weight M_n and the polydispersity *Đ* were determined by ^{1}H-NMR (in $CDCl_3$) and SEC (in THF) respectively.

**No.**	**H_2O sat. toluene** / g	**MG 2** / mL	**ε-CL** / g	**total H_2O content** / ppm	C_{oligo} / %	M_n / Da	***Đ***
2.1	1.26	2.2	0.82	338	92	6700	3.1
2.2	1.24	2.2	0.82	273	89	7000	3.1
2.3	1.24	2.2	0.81	319	91	6300	3.1
**Average**	**-**	**-**	**-**	**310**	**91**	**6700**	**3.1**

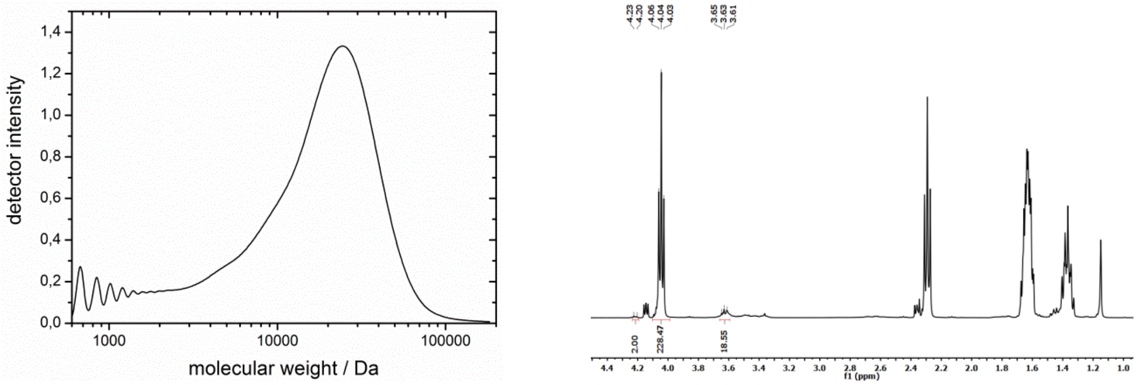

a) b)

Figure S6. a) SEC trace in THF and **b)** ^{1}H-NMR spectrum in $CDCl_3$ for the enzymatic polymerization of ε-CL with MG 2 (No 2.1).

6.3 Weights and Results for free CaLB-wt-lid

Table S5. Enzymatic polymerization with 1 mg CaLB-wt-lid: Weights of anhydrous toluene, water saturated toluene and anhydrous ε-CL; the corresponding conversion to oligomers C_{oligo}, the molecular weight M_n and the polydispersity $Đ$ were determined by ^{1}H-NMR (in $CDCl_3$) and SEC (in THF) respectively.

No.	anhydr. toluene / g	H_2O sat. toluene / g	ε-CL / g	total H_2O content / ppm	C_{oligo} / %	M_n / Da	$Đ$
3.1	0.72	2.53	0.83	285	21	1600	1.6
3.2	0.52	2.73	0.83	368	42	1000	1.3
3.3	0.51	2.73	0.85	368	45	1200	1.5
**Average**	**-**	**-**	**-**	**339**	**36**	**1300**	**1.5**

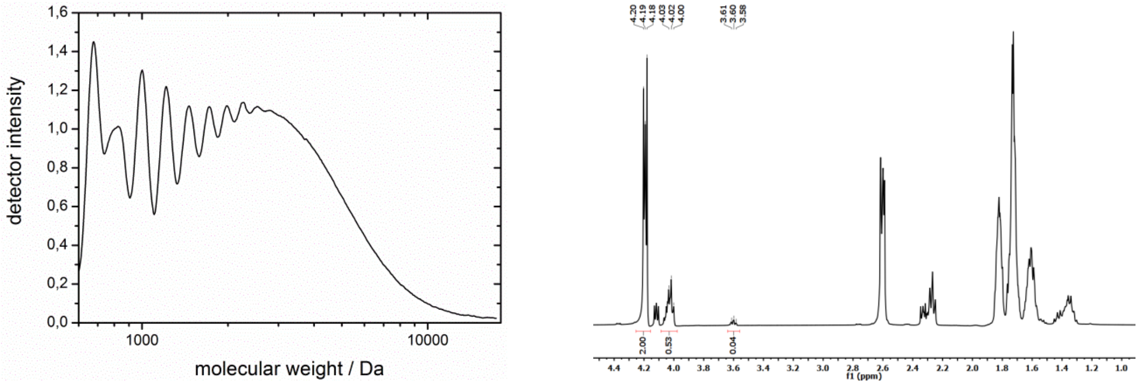

a) b)

Figure S7. a) SEC trace in THF and **b)** ^{1}H-NMR spectrum in $CDCl_3$ for the enzymatic polymerization of ε-CL with CaLB-wt-Lid (No. 3.1).

6.4 Weights and Results for MG 3 (CaLB-wt-lid)

Table S6. Enzymatic polymerization with MG 3 ([CaLB-wt-lid] = 0.45 wt/v): Weights of water saturated toluene, MG 3 and anhydrous ε-CL; the corresponding conversion to oligomers C_{oligo}, the molecular weight M_n and the polydispersity $Đ$ were determined by ^{1}H-NMR (in $CDCl_3$) and SEC (in THF) respectively.

No.	H_2O sat. toluene / g (%)	MG 3 / mL	ε-CL / g	total H_2O content / ppm	C_{oligo} / %	M_n / Da	$Đ$
4.1	1.26	2.2	0.79	363	19	1400	1.6
4.2	1.23	2.2	0.81	396	57	1300	1.8
4.3	1.19	2.2	0.82	396	60	1400	1.9
**Average**	**-**	**-**	**-**	**385**	**45**	**1400**	**1.8**

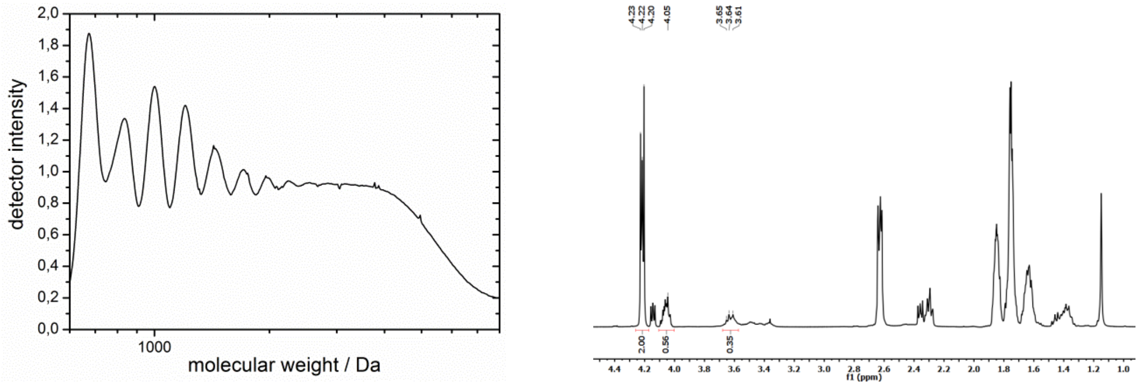

a) b)

Figure S8. a) SEC trace in THF and **b)** ^{1}H-NMR spectrum in $CDCl_3$ for the enzymatic polymerization of ε–CL with MG 3 (No. 4.1).

6.5 Weights and Results for free CaLB-degl-lid

Table S7. Enzymatic polymerization with 1 mg CaLB-degl-Lid: Weights of anhydrous toluene, water saturated toluene and anhydrous ε-CL; the corresponding conversion to oligomers C_{oligo}, the molecular weight M_n and the polydispersity *Đ* were determined by ^{1}H-NMR (in $CDCl_3$) and SEC (in THF) respectively.

**No.**	**anhydr. toluene** / g	**H_2O sat. toluene** / g	**ε-CL** / g	**total H_2O content** / ppm	C_{oligo} / %	M_n / Da	*Đ*
5.1	0.52	2.68	0.82	333	12	1400	1.5
5.2	0.52	2.70	0.79	335	11	1400	1.5
5.3	0.50	2.71	0.81	337	6	1300	1.4
**Average**	**-**	**-**	**-**	**335**	**10**	**1400**	**1.5**

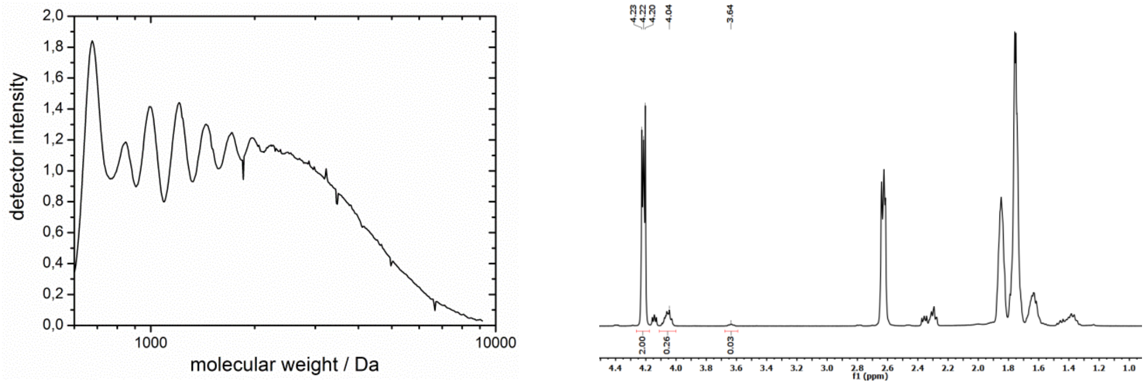

a) b)

Figure S9. a) SEC trace in THF and **b)** ^{1}H-NMR spectrum in $CDCl_3$ for the enzymatic polymerization of ε-CL with CaLB-degl-lid (No. 5.2).

6.6 Weights and Results for MG 4 (CaLB-degl-lid)

Table S8. Enzymatic polymerization with MG 4 ([CaLB-degl-lid] = 0.45 wt/v): Weights of anhydrous toluene, MG 4 and anhydrous ε-CL; the corresponding conversion to oligomers C_{oligo}, the molecular weight M_n and the polydispersity $Đ$ were determined by ^{1}H-NMR (in $CDCl_3$) and SEC (in THF) respectively.

**No.**	**anhydr. toluene** / g	**MG 4** / mL	**ε-CL** / g	**total H_2O content** / ppm	C_{oligo} / %	M_n / Da	$Đ$
6.1	1.24	2.2	0.82	323	3	1000	1.1
6.2	1.23	2.2	0.85	323	3	1000	1.1
6.3	1.23	2.2	0.81	323	4	1000	1.1
**Average**	**-**	**-**	**-**	**323**	**3**	**1000**	**1.1**

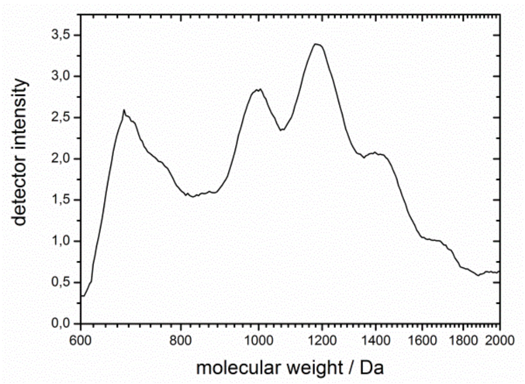

a)

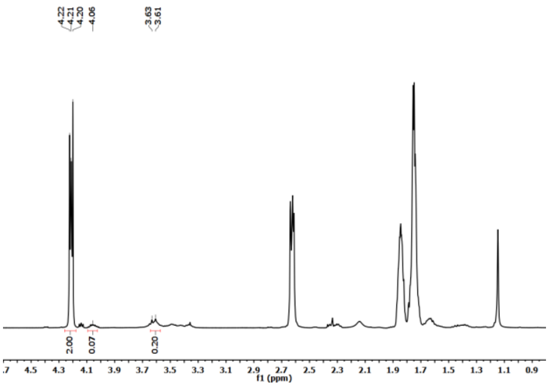

b)

Figure S10. a) SEC trace in THF and **b)** ^{1}H-NMR spectrum in $CDCl_3$ for the enzymatic polymerization of ε-CL with MG 4 (No. 6.2).

References

1. Engel, S.; Höck, H.; Bocola, M.; Keul, H.; Schwaneberg, U.; Möller, M. CaLB catalyzed conversion of ε-caprolactone in aqueous medium. Part 1: Immobilization of CaLB to microgels. *Polymers (Basel).* **2016**, *8*, 372, doi:10.3390/polym8100372.
